# Comparison between a Conventional Anti-Biofouling Compound and a Novel Modified Low-Fouling Polyethersulfone Ultrafiltration Membrane: Bacterial Anti-Attachment, Water Quality and Productivity

**DOI:** 10.3390/membranes10090227

**Published:** 2020-09-10

**Authors:** Norhan Nady, Noha Salem, Ranya Amer, Ahmed El-Shazly, Sherif H. Kandil, Mohamed Salah El-Din Hassouna

**Affiliations:** 1 Polymeric Materials Research Department, City of Scientific Research and Technological Applications (SRTA-City), Borg El-Arab City, Alexandria 21934, Egypt; 2 Department of Environmental Studies, Institute of Graduate Studies and Research, Alexandria University, Alexandria 21321, Egypt; nohasalem560@yahoo.com (N.S.); s.hassouna@gmail.com (M.S.E.-D.H.); 3 Environment and Natural Material Research Institute (ENMRI), City of Scientific Research and Technological Applications (SRTA-City), New Borg El-Arab, Alexandria 21934, Egypt; ranyaamer@yahoo.com; 4 Chemicals and Petrochemical Engineering Department, Egypt-Japan University of Science and Technology (E-JUST), Alexandria 21934, Egypt; ahmed.El-shazly@yahoo.com; 5 Department of Materials Science, Institute of Graduate Studies and Research, Alexandria University, Alexandria 21321, Egypt; s.kandil@usa.net

**Keywords:** biofouling, ultrafiltration, polyethersulfone, chlorine, membrane modification, low-fouling surface

## Abstract

In this work, the efficiency of a conventional chlorination pretreatment is compared with a novel modified low-fouling polyethersulfone (PES) ultrafiltration (UF) membrane, in terms of bacteria attachment and membrane biofouling reduction. This study highlights the use of membrane modification as an effective strategy to reduce bacterial attachment, which is the initial step of biofilm formation, rather than using antimicrobial agents that can enhance bacterial regrowth. The obtained results revealed that the filtration of pretreated, inoculated seawater using the modified PES UF membrane without the pre-chlorination step maintained the highest initial flux (3.27 ± 0.13 m^3^·m^−2^·h^−1^) in the membrane, as well as having one and a half times higher water productivity than the unmodified membrane. The highest removal of bacterial cells was achieved by the modified membrane without chlorination, in which about 12.07 × 10^4^ and 8.9 × 10^4^ colony-forming unit (CFU) m^−2^ bacterial cells were retained on the unmodified and modified membrane surfaces, respectively, while 29.4 × 10^6^ and 0.42 × 10^6^ CFU mL^−1^ reached the filtrate for the unmodified and modified membranes, respectively. The use of chlorine disinfectant resulted in significant bacterial regrowth.

## 1. Introduction

Proper pretreatment of the feed seawater for reverse osmosis (RO) helps to reduce microorganisms, thus protecting the RO membranes from fouling [1,2,3]. The ultrafiltration (UF) process is used as a pretreatment step; it serves as a barrier to remove components with a pore size larger than 100 nm, such as fine colloidal particles, bacteria, viruses, and larger molecules such as proteins [4,5,6]. The UF process has proved to be an effective alternative to conventional technologies in terms of both cost effectiveness and energy efficiency [2,7,8]. Moreover, according to environmental concerns, it is a good choice as it provides the production of higher quality brine with low levels of toxic chemicals and contaminants compared to conventional pretreatment technologies [3,9].

Generally, membrane systems are prone to several types of fouling depending on the type of foulant itself, e.g., inorganic or scaling fouling, particulate and colloidal fouling, organic fouling and biological fouling [10,11,12,13]. However, biological fouling is the most difficult to control in seawater desalination [13]. Biofilm formation consists of three major phases; induction, logarithmical growth, and plateau phases. The induction phase is the phase in which biofouling starts, with bacterial attachment to the membrane surface by weak physicochemical interactions [11,14,15]. The second phase is the logarithmical growth phase of the attached microorganisms. This phase is associated with extracellular polymeric substance (EPS) secretion and biofilm development [11,14]. The third phase is the plateau, where biofilm growth is limited by fluid shear forces. This phase is the detachment process, as bacteria tend to leave the biofilm for another part of the membrane surface due to an increase in population density and a lack of nutrients [11,12]. This stage of biofouling is more difficult control compared to earlier stages and is mainly affected by nutrients, bacterial growth rate, the mechanical stability of the biofilm, and the effective shear forces [13,14,15]. Generally, the rapid flux decline occurs at the early stage of biofilm formation due to the initial attachment and growth of microorganisms, followed by a gradual decay by the establishment of an equilibrium condition between the growth of biofilm, EPS production, and the detachment of cells [11,12,13,14,15].

There are three major strategies commonly used to control the biofouling phenomenon in membrane-based processes: chemical and physical pretreatment of the feed water to reduce nutrient availability [4,16,17,18], biocide usage for metabolic inactivation [17,19,20,21], and membrane modification to make the membrane less prone to biofouling [22,23,24,25]. The conventional method of dealing with the incidence of biofouling is to treat feed water with a biocide or disinfectant [18]. Chlorine remains the most commonly used disinfectant because of its availability, reasonable cost, and effectiveness [26,27,28]. Furthermore, the efficiency of chlorine can be improved with the use of coagulants to remove the suspended materials [29]. However, there are limitations to chlorine usage including the need for controlling pH, turbidity, and contact time [18,26]; also, hypochlorous acid, which is formed at lower pH values, is highly reactive and corrosive [30].

Most of the used membranes are very susceptible to chlorine degradation. Therefore, chlorination has to be followed by dechlorination in the pretreatment strategy when membranes are used for water treatment. It was reviewed that the oxidative nature of hypochlorite may have detrimental effects on polyethersulfone (PES) membranes such as high protein retention [27,30,31], polymer chain breakage, and consequent expansion of the membrane pore size [27,32,33,34], changes in membrane surface charge (hydrophilicity/hydrophobicity) [31,33,34], and the deterioration of the membrane’s mechanical strength [27,33].

Many bacteria can develop resistance against chlorine [28,35,36]. In addition, the removal of 99.9% of bacteria may not be sufficient to prevent their regrowth, as the surviving cells can multiply at the expense of biodegradable substances [37]. Moreover, the inactive biomass left in feed water after chlorination serves as a rich nutrient, resulting in a rapid bacterial growth rate [20,38]. Chlorine can also promote microbial regrowth by breaking down humic acids and producing assimilable organic carbon (AOC), which may act as a supportive nutrient for chlorine-resistant bacteria [18]. 

On the other hand, chlorine has environmental implications as it reacts with the organic matter of the feed water and produces various disinfection byproducts (DBPs) [39,40]. The types and concentrations of these DBPs depend on several factors such as the type and amount of disinfectant used, contact time, organic and inorganic contents, temperature, turbidity, and pH [13]. DBPs pose potential risks to human health and aquatic ecosystems when they are discharged in brine. Mediterranean seawater is particularly problematic as it usually contains high concentrations of bromide, which raise the risk of the formation of brominated DBPs that are more carcinogenic or mutagenic than their chlorinated analogs [41].

Another strategy used for fouling mitigation is the surface modification of ready-made membranes to acquire an effective anti-biofouling property [42,43]. PES is widely used for the preparation of UF membranes due to its excellent chemical resistance [44], good thermal stability, and mechanical properties [45]. PES membranes also show high flux and have a reasonable cost compared to other membrane materials. However, they are relatively hydrophobic, and their surfaces adsorb the components of the used fluid, which make them more susceptible to fouling [46]. Surface modifications of PES membranes are one of the current trends to control membrane fouling; they increase membrane surface hydrophilicity and consequently reduce the adsorption or adhesion of the different substances in feed water [47]. Surface modifications of PES membranes can be carried out in many ways, such as coating, blending, compositing, or grafting [24]. Several techniques can be used to initiate the grafting process, including chemical, photochemical, and high-energy radiation initiators [23,24,42,48], as well as enzymatic techniques [24].

Laccases are a group of oxidative enzymes whose exploitation as biocatalysts in the modification (grafting) of poly(ethersulfone) (PES) membranes represent a successful example of an environmentally friendly modification of PES membranes [49]. Phenols and aromatic or aliphatic amines are suitable substrates for laccase enzymes. Laccase-catalyzed reactions are proceeded by the monoelectronic oxidation of the substrate molecules to the corresponding reactive radicals that can then produce dimers, oligomers, and/or polymers [50,51]. 

Recently, a PES membrane was modified by the surface grafting of a brush-like hydrophilic polymer layer. This was achieved by enzyme-catalyzed grafting of an amine-bearing modifier, 3-aminophenol (3-AP), to obtain more hydrophilic PES membranes due to the presence of amine groups on the membrane surface. This method is known for its mildness and eco-friendliness as it can be carried out at room temperature, and uses only air as a source of oxygen and aqueous reaction medium, while no harsh chemicals are needed [47]. 

This study highlights an effective strategy to reduce biofouling in seawater desalination. It compares the effectiveness of membrane modification to reduce bacterial attachment, which is the initial step of biofilm formation, and the traditional strategy of using antimicrobial agents to kill bacteria cells in the seawater feed stream. The main aim of this study is to compare the efficiency of a conventional chlorination pretreatment step for feed water (seawater) with that of a modified PES UF membrane with brush-like oligomers of poly (3-AP) on its surface in terms of the UF membrane biofouling reduction, as well as comparing the environmental impacts of both strategies in terms of membrane performance and filtrate water quality. To the best of our knowledge, this is the first application study that compares the effect of membrane surface modification on the biofouling phenomenon compared to the traditional strategy of using antimicrobial agents that to mitigate biofouling in the membrane-based desalination process.

## 2. Materials and Methods

### 2.1. Materials

3-aminophenol (3-AP, C_6_H_7_NO), dichloromethane (DCM, CH_2_Cl_2_), sodium acetate (anhydrous, C_2_H_3_NaO_2_), acetic acid (C_2_H_4_O_2_), catechol (C_6_H_6_O_2_), and sodium thiosulfate pentahydrate (Na_2_S_2_O_3_·5H_2_O) were obtained from Sigma-Aldrich (Germany). All of them were at least 98% purity. A flat sheet of polyethersulfone (PES; 0.03 µm pore size) was purchased from Sterlitech (USA). Laccase from *Trametes versicolor* (>0.5 U·mg^−1^) was obtained from Fluka (Germany). Sodium hypochlorite solution (NaOCl, available chlorine 4%–5%) was purchased from Alpha Chemika (India). Sodium bisulfite (a mixture of NaHSO_3_ and Na_2_S_2_O_3_ powder) was obtained from Acros Organics (Belgium). Ethanol (analytical reagent grade) and *N,N*-diethyl-*p*-phenylenediamine 4 (DPD4) Palintest were purchased from Fisher (United Kingdom). Ferric chloride (FeCl_3_, anhydrous) was obtained from Oxford Laboratory (India). Luria–Bertani (LB) agar (Lennox) was obtained from Conda (Spain). Yeast extract was obtained from Bio Basic (Canada Inc., Canada). Peptone water medium (peptone 5.0, tryptone 5.0, sodium chloride 5.0) was purchased from Lab a Neogen Company (United Kingdom). Sodium phosphate monobasic, disodium hydrogen phosphate-2-hydrate, and potassium iodide (KI) were obtained from Riedel-de Haën (Germany). Soluble starch was purchased from Daejung (Korea). 

### 2.2. Methods

#### 2.2.1. Laccase Activity

Laccase activity was determined using catechol as a substrate, as previously described [46]. Briefly, the assay mixture contained 0.33 mL of 10 mM catechol, and 2.67 mL of 0.1 M sodium acetate buffer (pH 5), with 0.025 UmL^−1^ laccase. Catechol oxidation was monitored by following the increase in absorbance at 400 nm (ε = 26,000 M^−1^·cm^−1^) with a reaction time of 20 min. One unit of laccase activity is defined as the amount of enzyme required to oxidize 1 μmol of catechol per minute at 25 °C.

#### 2.2.2. Modification of PES Membrane Surfaces

Flat rectangular sheets of commercial PES membrane (200 × 200 mm, 0.03 µm pore size, (Sterlitech, USA) were cut into circles of 4.5 cm diameter to fit in a 50-mL Amicon filtration cell. The membrane modification was carried out as previously described [47,52]. The membrane circles were immersed in 40 mL of 0.1 M sodium acetate buffer (pH 5) containing equal volumes of 15 mM 3-AP and laccase enzyme (0.5 U∙mL^−1^). Air was bubbled through the solution for the purpose of good mixing and as a source of oxygen for the enzyme catalytic cycle (i.e., enzyme reactivation). The reaction was carried out for 30 min at room temperature (23 ± 2 °C). After completing the modification, the membrane circles were washed first by spraying with deionized water, followed by dipping in freshly boiled deionized water (95 ± 2 °C), and they were subsequently dried for 24 h in a desiccator.

#### 2.2.3. Seawater Sampling

**Figure 1:**
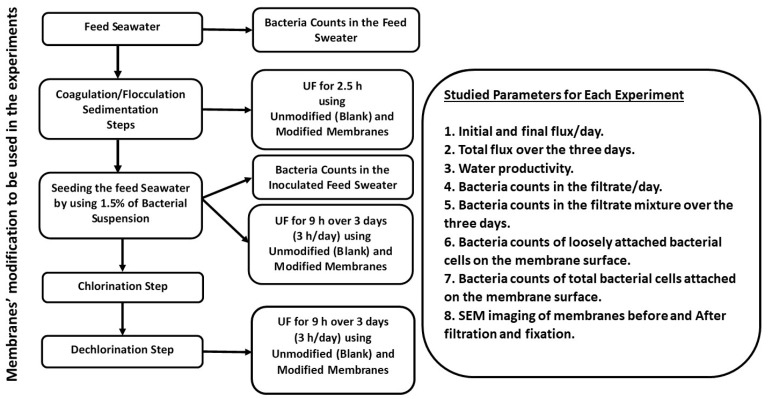
A schematic diagram of the experimental steps.

This study was conducted using Mediterranean seawater from the El Max region of west Alexandria, Egypt, in February 2018. Total dissolved salts (TDS), turbidity, and calcium content were measured immediately after sampling using standard methods [53]. The purpose of using seawater was to maintain the natural composition of the feed water used in the experiments. The feed seawater was stored at a controlled room temperature (20 ± 2 °C). Figure 1 shows a schematic diagram of the experimental steps, as described in the following sections.

#### 2.2.4. Preparation of the Feed Seawater

##### Pretreatment of the Feed Seawater by Coagulation

The collected seawater was pretreated to reduce the suspended matter in order to prevent membrane blockage by other types of fouling rather than biofouling, which was being investigated. Coagulation was carried out using ferric chloride (FeCl_3_) by the conventional standard jar test. A stock solution of FeCl_3_ (1000 mg·L^−1^) was prepared, 200 mL samples of seawater were placed in 250 mL beakers, and different concentrations of ferric chloride (2, 4, 6, 8, 10, 15, and 20 mg L^−1^) were added into the beakers. Samples were stirred for 1 min at 100 rpm followed by 20 min of slow mixing at 30 rpm [54]. Residual turbidity was determined as an indicator of performance, and the optimum dose of the coagulant was identified. The optimum dose of the FeCl_3_ was added to seawater and then kept for a week for sedimentation, and subsequently, the supernatant of clear water was taken.

##### Seeding of Seawater with Bacterial Load

The pretreated seawater was inoculated with various bacterial strains that are actually present in seawater in order to investigate the biofouling phenomenon within a relatively short time. Inoculum was prepared by adding 1 mL of seawater into 30 mL sterile Luria–Bertani (LB) broth (consisting of 0.5% tryptone, 0.5% peptone, 0.5% yeast extract, and 0.5% NaCl). The tubes were incubated in a shaker incubator at 30 °C and 150 rpm for five days. Then, the pretreated seawater was inoculated with 1.5% of this bacterial suspension (OD_680_ = 1.8) immediately before the UF experiment.

##### Disinfection of the Feed Seawater

The disinfection of the seawater was carried out using chlorine in the form of sodium hypochlorite (NaOCl) solution with 4%–5% available chlorine. Practically, only 3% of the available chlorine was determined by standardization using 0.01 N sodium thiosulfate (Na_2_S_2_O_3_) solutions. The standard method of iodometric titration [55] was used: 200 mL of chlorine solution was placed in a conical flask and 5 mL of glacial acetic acid was added to reduce the pH to between 3.0 and 4.0, followed by adding 1 g of potassium iodide, and the solution was mixed well. The potassium iodide solution was titrated against 0.01 N sodium thiosulfate solution until the yellow color of the liberated iodine almost faded away. Then, 1 mL of 1% starch solution indicator was added, producing a blue color, followed by titration again against 0.01 N sodium thiosulfate solution till the blue color disappeared; the total volume of titrant was measured, and total chlorine was determined in mg·L^−1^ from Equation (1):

Residual chlorine = A × N × 35.45 × 1000/mL sample taken(1)

where A is the mL of titrant for the sample and N is the normality of sodium thiosulfate. 

In this work, a stock solution of sodium hypochlorite (1000 mg L^−1^) was freshly prepared. A chlorine dose of 6 mg L^−1^, which was prepared from this stock solution, was added to the inoculated pretreated feed water (pH 6) and was kept for 90 min contact time in the dark at room temperature (23 ± 3 °C). Then, the free residual chlorine was measured using the standard DPD colorimetric method. 

##### Dechlorination Process

Sodium bisulfite was added as a dechlorinating agent for the removal of any residual chlorine before UF to protect the membrane from deterioration by chlorine; a stock solution of sodium bisulfite (100 mg L^−1^) was prepared using sterile distilled water. Excess sodium bisulfite was used to confirm the complete removal of chlorine (each 5 mg L^−1^ of sodium bisulfite was added to remove 1 mg L^−1^ of residual chlorine), kept for 30 min contact time at a room temperature of 23 ± 2 °C. Then, residual free chlorine was measured using the DPD colorimetric method; a chlorine standard curve was established by the preparation of different known concentrations of chlorine (0.05, 0.2, 0.5, 0.8, 1, 2, and 3 mg L^−1^) in 10 mL sterilized distilled water. The concentration of residual chlorine was measured by adding DPD tablets into 10 mL of solution and shaking for 2 min. The intensity of the produced red color was measured using a Vis-spectrophotometer at 515 nm wavelength, and the concentration of residual chlorine was determined in mg L^−1^ using the prepared chlorine standard curve. 

#### 2.2.5. Ultrafiltration (UF) 

A dead-end stirred filtration cell (Millipore, Amicon Model 8050, 13.4 cm^2^ active filtration area) was used at a constant pressure of 1 bar and 200 rpm stirring at 23 ± 2 °C. Unmodified and modified PES membranes were used in the filtration of different conditions of feed seawater. Two different feed seawater types, in terms of pretreatment steps, were used: one pretreated by coagulation only, without the chlorination step, and the other pretreated by coagulation followed by chlorination and dechlorination steps. Then, the pretreated feed seawater samples were seeded with a bacterial suspension and filtered for 9 h over three days (3 h/day) of filtration time. UF was also carried out for the pretreated feed seawater without bacterial loading for 2.5 h of filtration time in order to investigate the membrane permeability in the absence of bacterial cells.

First, the filtration cell was immersed in 70% ethanol overnight, and then it was washed three times with sterilized deionized water to remove any traces of ethanol. The membranes were also washed three times with sterilized deionized water before the experiment, and then UF was carried out. At the end of each day, the membrane was placed in 50 mL of initial pretreated seawater without bacterial suspension to avoid any damage caused by drying. The experiment for each tested condition was performed three times; a new membrane was used in each experimental assay. The microbiological results were normalized and their average was taken.

##### Water Flux 

The pure water flux was determined at the start and end of each day using Equation (2):

(2) $$J_{w}\;=\;\frac{Q}{\Delta t\cdot A}$$

where J_w_ = water flux (m^3^·m^−2^·s^−1^), *Q* is the volume of permeate collected (m^3^), Δ*t* is the sampling time (s), and *A* is the membrane area (m^2^) [47].

##### Water Productivity

The volume of the output of filtrate from the membrane was determined in m^3^·h^−1^ at the end of each day for three days.

#### 2.2.6. Bacterial Count 

Bacterial growth was counted in the initial feed seawater and the filtrate produced each day as well as the filtrate mixture produced. Standard plate counts were used by plating 100 µL of suitable serially diluted bacterial suspension in phosphate buffer solution (PBS) pH 7, ranging from 10^−1^ to 10^−5^, on LB agar in three replicates, followed by incubation at 30 °C overnight. The number of separate colonies was recorded as a colony-forming unit (CFU) mL^−1^ [56].

Moreover, at the end of the experiment, the membrane was cut into two identical halves; one half was placed in 30 mL PBS and the bacteria attached on the membrane surface were determined twice; the first bacterial count was determined immediately for the loosely attached bacteria by gently handshaking for a minute, and then the count was determined again for the total bacterial cells attached on the membrane surface using mechanical shaking after incubation overnight in a shaker incubator at 150 rpm, 30 °C. The bacterial counts were determined as the CFU m^−2^ of the membrane surface. 

#### 2.2.7. Scanning Electron Microscope Imaging

Both unmodified and modified membrane surfaces, after UF of both pretreated, inoculated feed seawater without the chlorination step and pretreated, inoculated feed seawater with chlorination and dechlorination steps, were imaged using a JeolJsm 6360 LA scanning electron microscope (SEM, JEOL Ltd., Tokyo, Japan). After the experiment, the membranes were cut using a very sharp blade and were preserved in a fixer composed of 0.3% glutaraldehyde, 5% formaldehyde in phosphate buffer (pH 7.2), and serially dehydrated in modified ethyl alcohol [57]. Moreover, the formed layers on both unmodified and modified PES membranes, after UF of pretreated feed seawater with neither the chlorination step nor bacterial loading and after UF of pretreated feed seawater with the chlorination step and without bacterial loading, were imaged. All surfaces were coated with Au before imaging. A voltage of 20 KV and a resolution of 1280 × 960 pixels were used.

#### 2.2.8. Atomic Absorption Spectroscopy Analysis

Atomic absorption spectroscopy (Shimaddzu AA-7000, Tokyo, Japan) was used for the analysis of the layers formed on both the unmodified and the modified PES UF membrane surfaces after filtration of the two types of feed seawater, without bacterial loading (feed seawater pretreated without the chlorination step and feed seawater pretreated with chlorination and dechlorination steps), at a constant pressure of 1 bar and 200 rpm, at 23 ± 2 °C, for 2.5 h of filtration time.

## 3. Results and Discussion

### 3.1. Chemical Analysis of the Used Seawater

A chemical analysis of the seawater showed a high calcium content (483.36 ± 8.92 mg L^−1^) due to the winter season, as previously reported [58]. The turbidity was determined as 3.17 Nephelometric Turbidity Units (NTU). The total dissolved salts (TDS) concentration was 27.2 ppt (ng/L), which is less than the average for Mediterranean seawater [59] because of the proximity of the El-Mahmoudiyah canal outfall to the sampling point.

### 3.2. Membrane Characterization

The membrane characterization was previously performed and published [47], and the obtained results are briefly presented as follows: Thermogravimetric Analyses (TGA) show that the rate of decomposition of the backbone of the modified membranes is somewhat slower than that of the blank membrane. As shown at 800 °C, only 38 wt% of the modified membrane remained, whereas only 15 wt% remained of the blank membrane. Moreover, Differential Scanning Calorimetry (DSC) analysis revealed that the glass transition temperature of the blank PES membrane was 226 °C, and it decreased very slightly upon modification to 224 °C. X-ray diffraction (XRD) analysis shows the effect of the amorphous structure of poly(3-AP) on the intensity of the characteristic peak of the blank membrane; it is proposed that the addition of poly(3-AP) may contribute to the increase in the flux of the modified membranes. The tensile strength test of the membranes showed a very slight decrease in the tensile strength of the blank membranes. However, the modified membranes at a high grafting yield showed slightly stronger mechanical properties than the blank membrane. The Raman spectra of the modified membrane confirm the presence of amine groups on the membrane surface. Scanning Probe Microscope (SPM) images show the formation of a brush-like modifying layer of poly (3-AP). Furthermore, the Nuclear Magnetic Resonance (NMR) integration results of the analyzed peaks do not favor a particular structure. The proposed structure of the formed poly(3-AP) layer is shown in Supplementary Figure S1. The water flux of the most modified membranes increased up to 35% relative to the blank (unmodified) membrane, and an up to 90% reduction in protein adsorption was obtained. In general, this modification does not harmfully affect the bulk properties of the original blank membrane.

### 3.3. Pretreatment of the Feed Seawater (Coagulation–Flocculation)

Coagulation and flocculation are important pretreatment processes for the removal of colloidal particles responsible for the turbidity of seawater [13]. The destabilization of colloidal particles is usually carried out by adding coagulants followed by the clotting of the resulting unstable colloidal particles, which are then removed from water by sedimentation [60]. In this work, coagulation was carried out by the standard jar test using ferric chloride (FeCl_3_) due to its proven performance as a coagulant in water treatment plants [61]. The addition of FeCl_3_ resulted in the rapid removal of turbidity as a result of the neutralization of the negatively charged particles with different cationic species produced from the hydrolysis of ferric chloride in water, leading to the destabilization of such particles and subsequently flocculation (Supplementary Figure S2). Maximum turbidity removal was about 82.6% at 8 mg/L of FeCl_3_ (Supplementary Figure S3). However, when high concentrations of FeCl_3_ were used, lower turbidity removal was obtained due to competition between the re-conformation rate of negatively charged particle networks and the collision rate of destabilized colloids [61,62].

### 3.4. Ultrafiltration Process

#### 3.4.1. Water Flux

##### Pretreated Feed Seawater without Bacterial Loading

**Figure 2:**
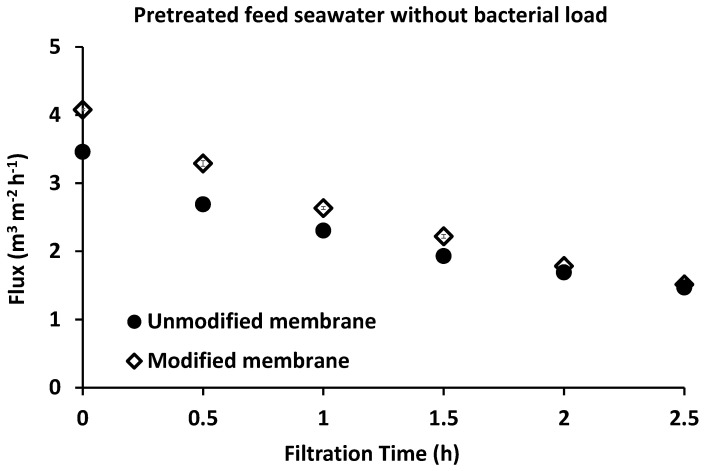
The flux of pretreated seawater with neither bacterial load nor the chlorination step. Three samples were tested for both the unmodified and modified membranes. Reference conditions: pressure of 1 bar, 23 ± 2 °C at 200 rpm stirring for 2.5 h filtration time. Unmodified membrane (black filled circle) and modified membrane (black unfilled diamond).

**Figure 3:**
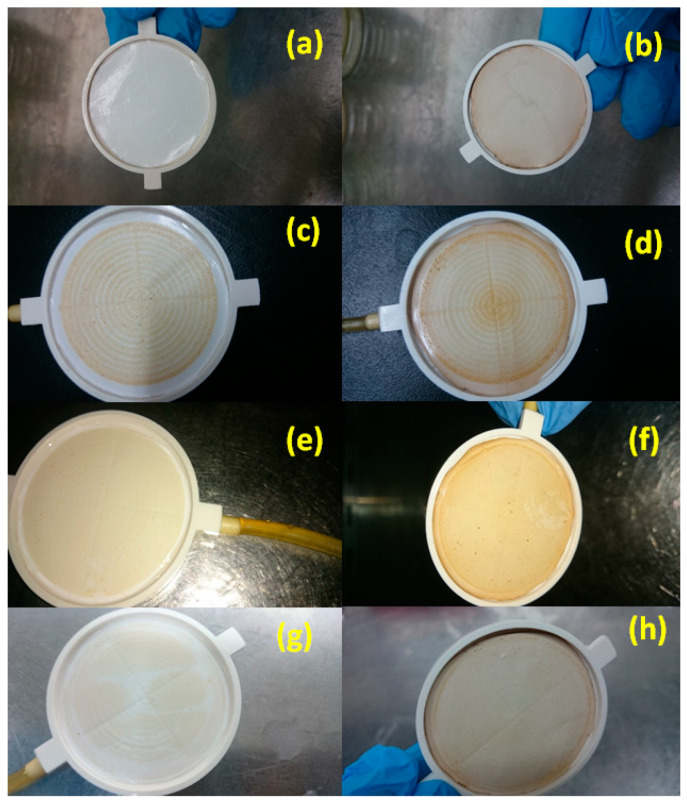
Photos of unmodified membrane before (**a**) and after (**c**) filtration of pretreated seawater without bacterial loading, and modified membrane before (**b**) and after (**d**) filtration of pretreated seawater without bacterial loading. Photo of unmodified membrane after filtration of pretreated, inoculated seawater without chlorination (**e**) or with the chlorination (**g**) pretreatment step, and photo of modified membrane after filtration of pretreated, inoculated seawater without chlorination (**f**) or with the chlorination (**h**) pretreatment step. Reference conditions: 1 bar, 23 ± 2 °C and 200 rpm stirring for 2.5 h filtration time.

**Figure 4:**
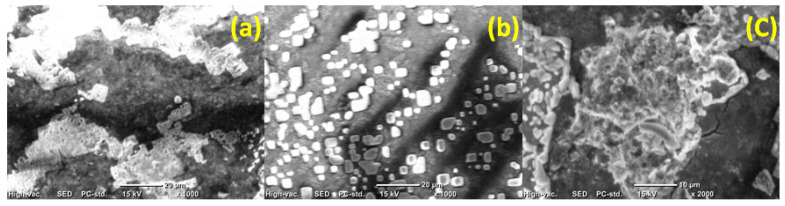
SEM images of unmodified ((**a**), ×1000) and modified ((**b**), ×1000) polyethersulfone (PES) membranes after filtration of pretreated seawater with neither bacterial loading nor the chlorination step, and modified ((**c**), ×2000) PES membranes after filtration of pretreated seawater without bacterial loading but after the chlorination step. Reference conditions: 1 bar, 23 ± 2 °C and 200 rpm stirring for 2.5 h filtration time.

A UF experiment was carried out using pretreated seawater with neither bacterial loading nor the pre-chlorination step in order to investigate the effect of other seawater contents, which can affect membrane performance. As shown in Figure 2, the water flux reduced over time for both unmodified and modified membranes to less than half its initial value (unmodified, 57.5% reduction; modified, 62.9% reduction). Observation of the membrane before and after filtration showed the formation of a colored layer that precipitated on the membrane surface (Figure 3) (unmodified PES, c; modified PES, d). SEM images of this formed layer showed salt precipitation. The presence of calcium was proposed due to its high content in the raw feed seawater (483.36 ± 8.92 mg L^−1^) and was confirmed by atomic absorption analysis. However, there may be other salts/dissolved materials that were precipitated on the membrane surface. It should be noted that, in desalination plants, the inlet feed is usually diluted to reduce the water salinity to around 15,000–20,000 ppm to minimize salt precipitation on the membrane surface. Calcium ions have a negative impact on the membrane flux by altering the surface chemistry through interaction with foulant molecules, such as natural organic materials (NOM) [11,63,64]. Calcium can also link two negatively charged functional groups together to form intermolecular complexes; when the linkage happens between two humic acid molecules, a gel layer of macromolecules can be formed through this intermolecular bridging, and it becomes more compact and cohesive by the cross-linking effect of calcium [64]. As shown in Figure 4, scanning electron microscope images showed that most of the formed layers appeared as separate crystals on the modified membranes, whereas they appeared as a packed gel layer on the unmodified PES membrane. The pre-chlorination step of the feed seawater did not make a significant change to the general performance of both unmodified and modified membranes (i.e., only a fluctuation up to 6%).

##### Pretreated Feed Seawater with Bacterial Loading

The bacterial count of the seawater sample was determined as 1600 CFU mL^−1^. In order to investigate the biofouling phenomenon within a relatively short time, the pretreated seawater was inoculated with a bacterial load of various bacteria strains that are naturally present in seawater. The seawater was seeded by a 1.5% bacterial suspension of OD_680_ = 1.8 and was filtered using a dead-end stirred filtration cell at a constant pressure of 1 bar at 23 ± 2 °C and 200 rpm stirring for 9 h (3 h × 3 days) filtration time. PES membranes of 0.03 µm pore size were used in the filtration of pretreated (i.e., by the coagulation step as described in the previous section) inoculated feed seawater, with or without the pre-chlorination step. The biofouling phenomenon and biofilm formation on the PES membrane surface with the consequential effect on membrane performance was studied. Membrane performance was evaluated by determining: (1) the initial and final flux of the membrane, (2) water productivity (volume of filtrate), (3) bacterial count of the filtrate on each day, (4) bacterial count of the filtrate mixture produced over three days, (5) bacterial count on the membrane surface and (6) SEM imaging of the membrane surface.

**Figure 5:**
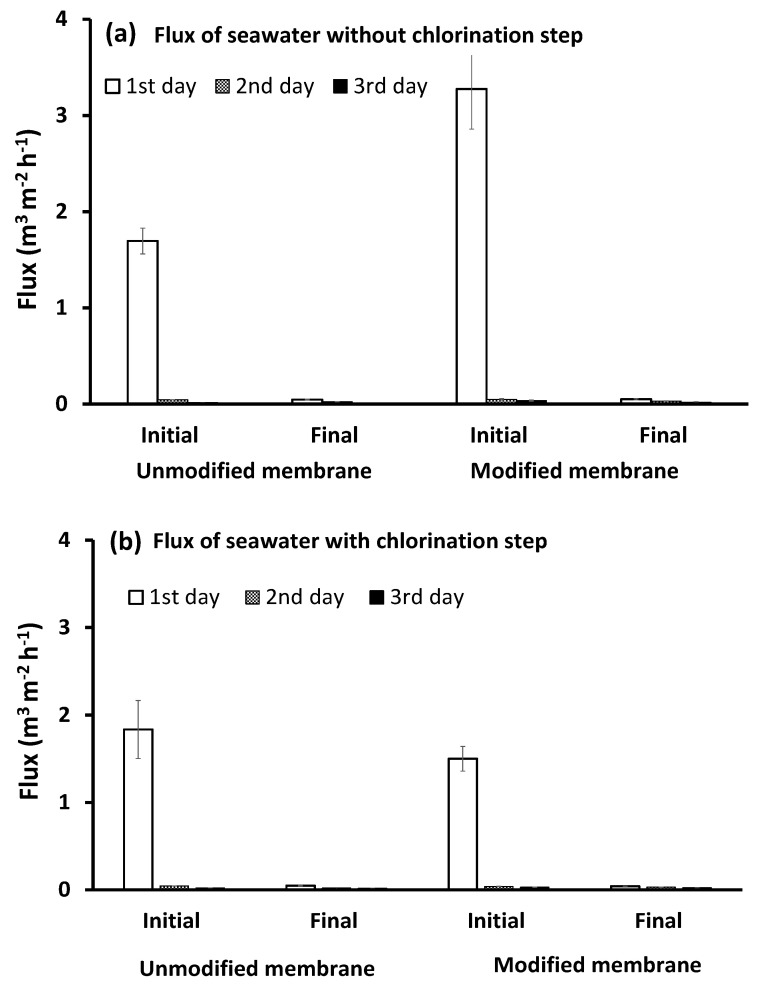
Initial and final flux of both the unmodified and modified PES membranes in two cases: bacterial loaded feed seawater without chlorination (**a**) or with the chlorination (**b**) pretreatment step. Reference conditions: pressure of 1 bar at 23 ± 2 °C and 200 rpm stirring for 9 h (3 h × 3 days) filtration time.

As shown in Figure 5, on the first day of filtration of the pretreated, inoculated feed seawater, without the chlorination step, the initial flux of the unmodified membrane was significantly reduced, and only about 43% of its value was maintained relative to seawater without bacterial loading. Meanwhile, the modified membrane was able to maintain about 76.3% of its initial flux relative to seawater without bacterial loading. The modified membrane had a higher initial flux compared to the unmodified one, which can be attributed to the presence of free polar groups of brush-like oligomers formed on the membrane surface, as previously presented and shown in Supplementary Figure S1 [47], which increased both its hydrophilicity (the static water contact angle of the unmodified and modified PES are 75.9 ± 2° and 41.2 ± 1.7°, respectively) and the repellence of bacteria. Both effects can facilitate water permeation. The flux declined rapidly to reach about 1% of its initial flux for both unmodified and modified membranes by the end of the first day (3 h filtration). This rapid flux decline can be correlated with two main effects. 

The first effect is concentration polarization, which resulted from the accumulation of larger solutes (such as calcium crystals, as illustrated in the previous section) that were rejected and retained at the membrane surface. As they could not diffuse back to the bulk solution, they caused a concentration gradient above the membrane surface and created an osmotic back pressure that reduced the effective transmembrane pressure of the system [11,64].

The second factor responsible for the rapid flux decline was the early attachment and proliferation of bacterial cells maintained on the membrane surface [11,17,65,66]. Bacterial cells colonized the membrane through the reversible and irreversible attachment of bacteria’s surface via electrostatic and hydrophobic interactions [17] as the first step of biofilm formation. Moreover, depositions of bacterial cells on the membrane surface formed hydraulic resistance, which resulted in additional concentration polarization as bacterial cells affected the porosity and pore size distribution on the membrane surface, resulting in the precipitation of salts within membrane pores [20,65].

On the other hand, when the pretreated, inoculated seawater was exposed to chlorination and dechlorination pretreatment steps, it was observed that the chlorination did not cause evident changes in the initial flux of the unmodified membrane, as it decreased to about 53% of its value relative to the feed seawater without bacterial loading. Meanwhile, the chlorination step resulted in a reduction in the modified membrane flux to about 36.7% of its value relative to the case of using feed seawater without bacterial loading, and an even greater reduction compared to the pretreated, inoculated seawater without chlorination (45.8%). There is no obvious explanation for the effect of chlorination on the modified layer; however, SEM images of the salt layer formed on the membrane surface, when UF was carried out using pretreated seawater without bacterial loading, showed a difference in the shape of the formed layer in the presence or absence of chlorine. When the feed water was pretreated without the chlorination step, a continuous gel layer was formed on the unmodified membrane surface (Figure 4a), while clearly separated crystals were formed on the modified membrane surface (Figure 4b). However, when chlorine was used, the salt layer formed on the modified membrane (Figure 4c) was similar to that formed on the unmodified membrane. The significant flux reduction in the modified membrane after the filtration of pretreated, inoculated seawater with chlorination and de-chlorination pretreatment steps may be explained by the presence of dead biomass in the feed water which represented a high content of NOM, which could interact with calcium ions and form a thick, packed gel layer that affects membrane permeability. Furthermore, this condensed gel layer could be easily consumed by bacterial cells resulting in a higher bacterial growth rate. However, the effect of the chlorination–dechlorination pretreatment steps on the modified membrane structure and the mechanisms by which these two different layers were formed in the presence or the absence of chlorine require further investigation. The very low flux observed on the second and third days of filtration was a common trend for both the unmodified and modified PES membranes in the two cases of pretreated, inoculated chlorinated or non-chlorinated seawater. However, the rate of flux decline was more gradual, which can be related to the equilibrium condition between biofilm growth, EPS production, and biofilm loss (cell detachment) caused by hydrodynamic shear at the solution–biofilm interface [17,66].

#### 3.4.2. Water Productivity

**Figure 6:**
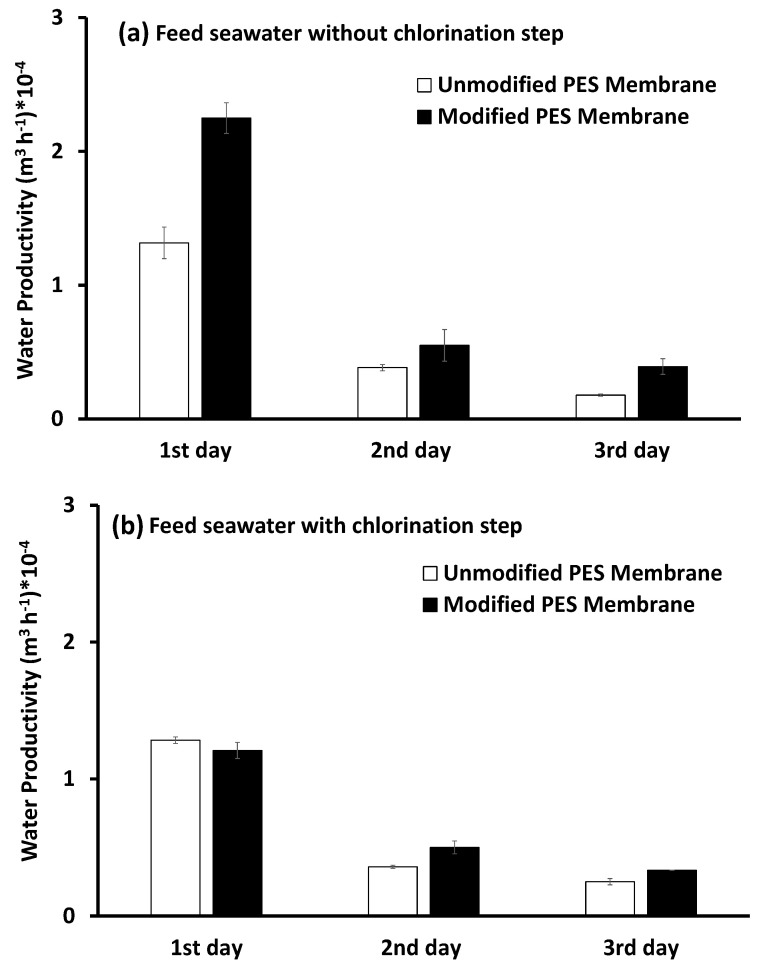
Water productivity (m^3^ h^−1^) of both the unmodified and modified PES membranes in two cases: bacterial loaded feed seawater without chlorination (**a**) or with the chlorination (**b**) pretreatment step. Reference conditions: pressure of 1 bar at 23 ± 2 °C and 200 rpm stirring for 9 h (3 h × 3 days) filtration time.

The filtrate volume per unit of time is expressed as water productivity (m^3^·h^−1^), as shown in Figure 6. The highest productivity was recorded on the first day for the different testing conditions. Meanwhile, the productivity was greatly decreased on the second and third days of filtration (about 73 and 84%, respectively) as a consequence of flux decline. The largest volume of the filtrate was produced when the modified membrane was used to filter pretreated, inoculated seawater, without the chlorination step (water productivity was almost one and a half times the water productivity of the unmodified membrane). When chlorine was used, the productivity of the modified PES membrane was reduced due to flux decline, as mentioned in the previous section.

#### 3.4.3. Bacterial Counts

##### Bacterial Counts in the Pretreated Inoculated Feed Seawater over Three Days

**Figure 7:**
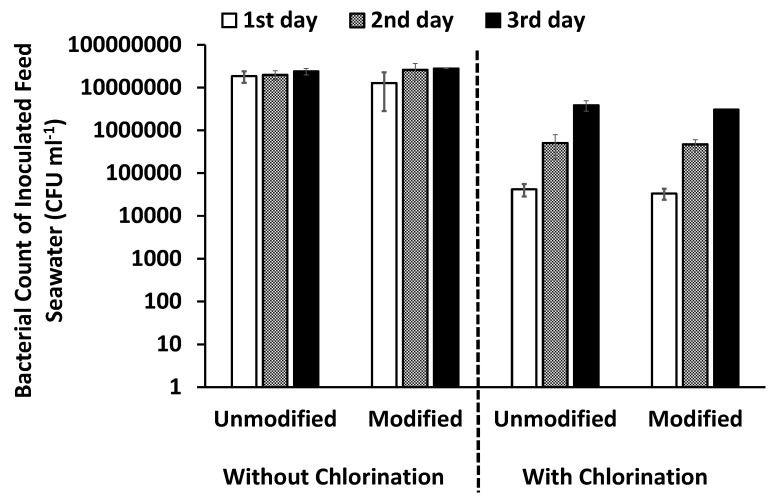
Bacterial count in pretreated, inoculated chlorinated and non-chlorinated feed seawater.

Chlorine is usually added to control bacterial growth in most water treatment/desalination plants; the effect of the chlorination step on the feed water was investigated by counting the bacterial cells in pretreated, inoculated feed seawater over three days. The feed seawater used for the experiments was freshly prepared on the first day and then kept at 4 °C overnight to be used on the second and third days. With the chlorination pretreatment step for feed water, the rate of bacterial growth increased rapidly over the three days relative to the bacterial growth of feed water that was not chlorinated, as shown in Figure 7. This high growth rate may be attributed to the presence of a rich nutrient supply of inactive biomass (dead bacteria) in the feed water [20,38]. This means that chlorine is not the optimum choice to control bacterial growth, even if it is efficient to remove most of the bacteria, as the surviving bacteria can undergo a rapid regrowth.

###### Bacterial Counts in the Filtrate Water over Three Days

**Figure 8:**
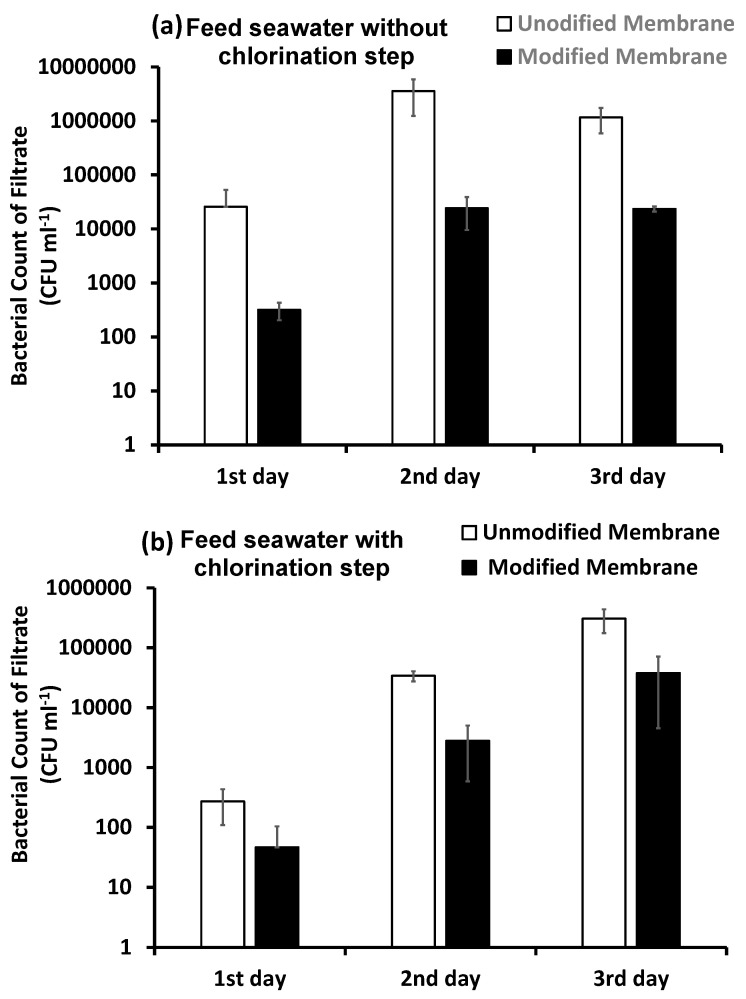
Bacterial count of the ultrafiltration (UF) filtrate for pretreated, inoculated feed seawater without chlorination (**a**) or with the chlorination (**b**) pretreatment step. Reference conditions: 1 bar, 23 ± 2 °C and 200 rpm stirring for 9 h (3 h × 3 days) filtration time.

Since UF is commonly used to remove fine colloidal particles, bacteria, viruses and large molecules such as proteins [5], both unmodified and modified membranes showed a high bacterial removal efficiency under the different testing conditions. On the first day of filtration of the pretreated, inoculated feed seawater (Figure 8), about 99.8% of total bacterial cells were removed by the unmodified membrane (retained on the membrane), while about 0.2% were permeated with the filtrate (2.575 × 10^4^ CFU mL^−1^). The highest removal of bacterial cells was achieved by the modified membrane, in which about 99.99% of total feed bacterial cells were retained on the membrane surface, while only 0.01% of feed bacteria (0.318 × 10^4^ CFU mL^−1^) reached the filtrate. This high bacterial rejection confirms the antifouling ability of such a modification, as illustrated in a previous work [47]. The antifouling mechanism of the modified membrane is based on steric hindrance and the osmotic effect of the hydrated brush-like polymer layer, which keeps bacterial cells as well as macromolecules (nutrients for bacteria) at a distance from the membrane surface [50]. On the second day of filtration, the bacterial cells on the membrane surface began to metabolize and secrete extracellular polymeric substances as the first step of biofilm formation [11,14,15,17]. For this, the filtrate on the second day recorded the highest bacterial count for both the unmodified membrane (355 × 10^4^ CFU mL^−1^) and modified membrane (2.41 × 10^4^ CFU mL^−1^); however, the counted bacterial cells in the filtrate of the modified membrane were much lower than those in the filtrate of the unmodified membrane.

This phenomenon can be attributed to the logarithmical growth phase. Meanwhile, bacterial growth may be promoted by the presence of calcium salts. Calcium not only causes flux decline, as discussed before, but also plays a vital role in bacterial biofilm formation. The calcium ion was reported as a universal messenger, transmitting signals from the cell surface to the interior of the cell [67,68]. Calcium signaling is regulated by calmodulin, which is a calcium-modulating protein that controls cell proliferation, programmed cell death, and autophagy [69]. Moreover, calcium was assigned in specific and non-specific interactions between cells and the localized surface, in which calcium-binding proteins are often involved in bacterial adhesion to a surface. This binding is important for cell–cell aggregation. In addition, calcium is also recorded as an ionic cross-bridging molecule for negatively charged bacterial polysaccharides [68].

On the third day of filtration, the bacterial count recorded in the filtrate of the unmodified membrane was lower than that on the second day. This may be explained as the bacterial growth reaching the plateau phase, in which the biofilm growth phase was limited by the “detachment process” of the fluid shear forces. This phase may be attributed to the increase in population density and the lack of nutrients in the biofilm, and because the bacterial attachment to the membrane is limited [11,14,15]. Another reason for the inability of bacteria to reach the filtrate of the unmodified membrane is the complete blockage of most membrane pores. Regarding this, the bacterial cells were forced to settle on the membrane surface. Meanwhile, in the case of the modified membrane, the bacterial counts of the filtrate on the second and third days were approximately the same. This can be explained by the incomplete blockage of membrane pores. The modified membrane had available spaces for bacterial attachment. This observation was confirmed by SEM images, as will be discussed in the following section.

**Figure 9:**
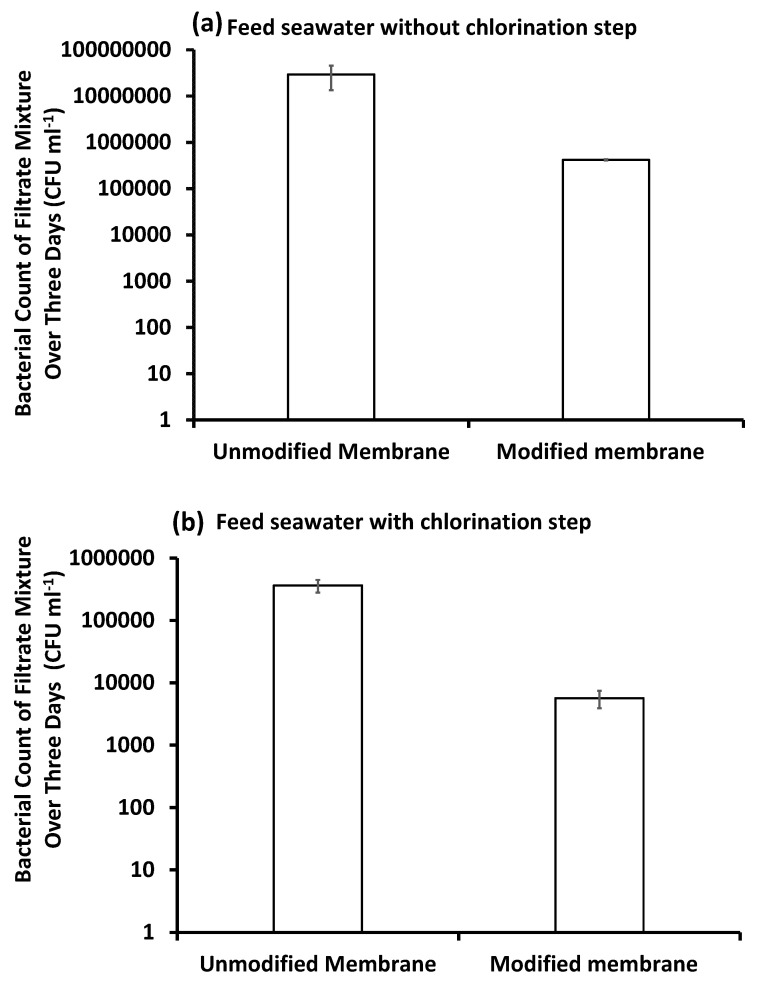
Bacterial count of the filtrate mixture produced after three days of filtration using pretreated, inoculated feed seawater without chlorination (**a**) or with the chlorination (**b**) pretreatment step. Reference conditions: 1 bar, 23 ± 2 °C, and 200 rpm stirring for 9 h (3 h × 3 days) filtration time.

When chlorine was applied in the pretreatment, it removed about 99.6% of the total bacterial cells in the feed water (Figure 9). On the first day of filtration, the unmodified membrane removed about 99.3% of the bacterial cells that remained in the feed water after the chlorination step, and only 0.7% of bacterial cells reached the filtrate. Bacterial counts in the filtrate increased by the second and third days of filtration to reach about 306.5 × 10^3^ CFU mL^−1^ by the end of the third day. This was attributed to the growth of bacterial cells on the membrane surface due to dead biomass and calcium ions, as mentioned before. Although the modified membrane showed a significant flux decline in the presence of chlorine, as illustrated in the previous section, it was efficient in removing most of the total bacteria remaining in the feed seawater after the chlorination step over the three days of filtration. In addition, the modified membrane showed the lowest bacterial counts recorded for filtrate mixtures of both chlorinated and non-chlorinated feed seawater.

##### Bacterial Count on the Membrane Surface

**Figure 10:**
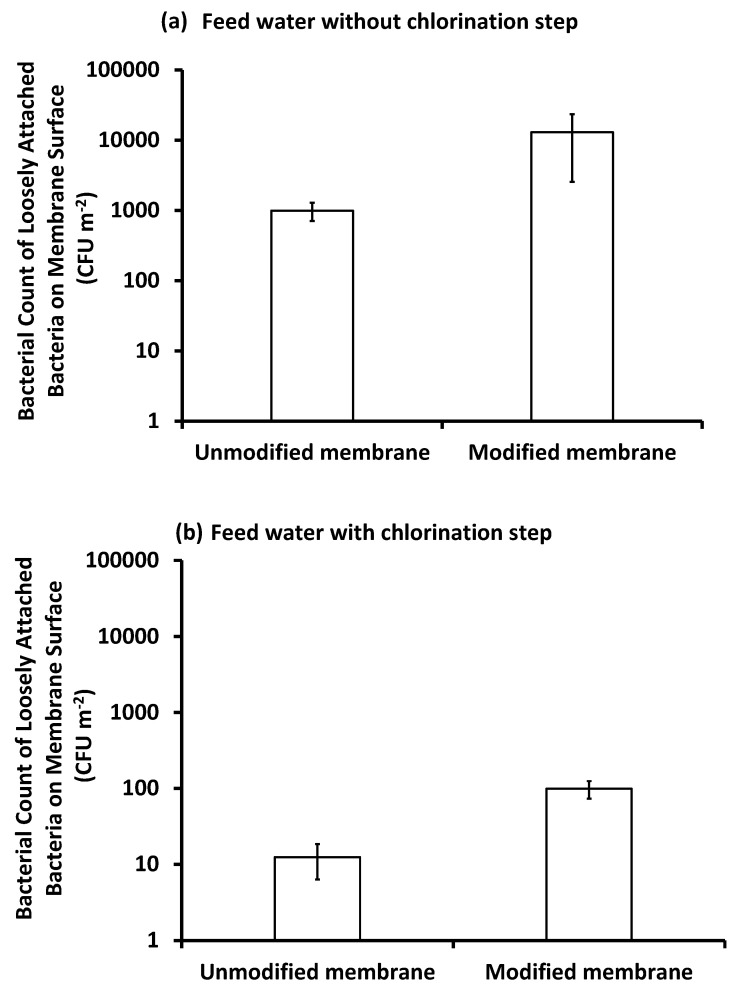
Bacterial count of loosely attached bacteria on the surface of unmodified and modified PES membranes after three days of filtration under different conditions of feed water: feed water pretreated without the chlorination step (**a**), and feed water pretreated with the chlorination step (**b**) using hand shaking for 1 min. Reference conditions: 1 bar, 23 ± 3 °C at 200 rpm stirring for 9 h (3 h × 3 days) filtration time.

**Figure 11:**
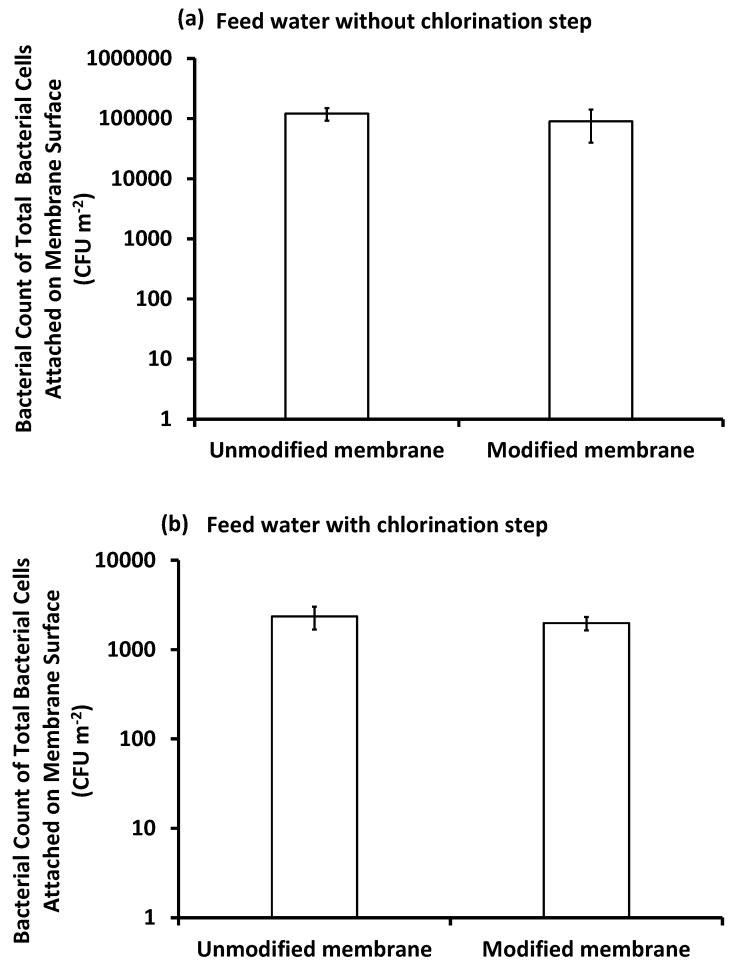
Bacterial count of total bacteria attached on the surface of the unmodified and modified PES membranes after three days of filtration under different conditions of feed water: feed water pretreated without the chlorination step (**a**), and feed water pretreated with the chlorination step (**b**) using mechanical shaking for 24 h. Reference conditions: 1 bar, 23 ± 2 °C at 200 rpm stirring for 9 h (3 h × 3 days) filtration time.

As shown in Figure 10 and Figure 11, the bacteria that were removed from the modified membrane surface after the filtration of inoculated feed seawater, pretreated with or without chlorine, after gentle handshaking for 1 min, were more than those removed from the unmodified membrane. Meanwhile, the total bacterial cells removed from the unmodified membrane surface after mechanical shaking for 24 h were more than those removed from the modified membrane surface. This can be explained as a looser attachment of cells to the modified membrane compared to the unmodified membrane. This, in fact, confirms the antifouling effect of the modified membrane as it can keep bacteria at a distance from the membrane surface [47]. Meanwhile, the bacteria on the unmodified membrane were more closely attached as it is more hydrophobic and hence more favorable for bacterial attachment [17]. This was shown by the thick layer of biofilm and EPS secretion, as evidenced by SEM images. Based on this result, we can say that, after routine washing, the modified membrane can retain its normal flux and performance.

**Figure 12:**
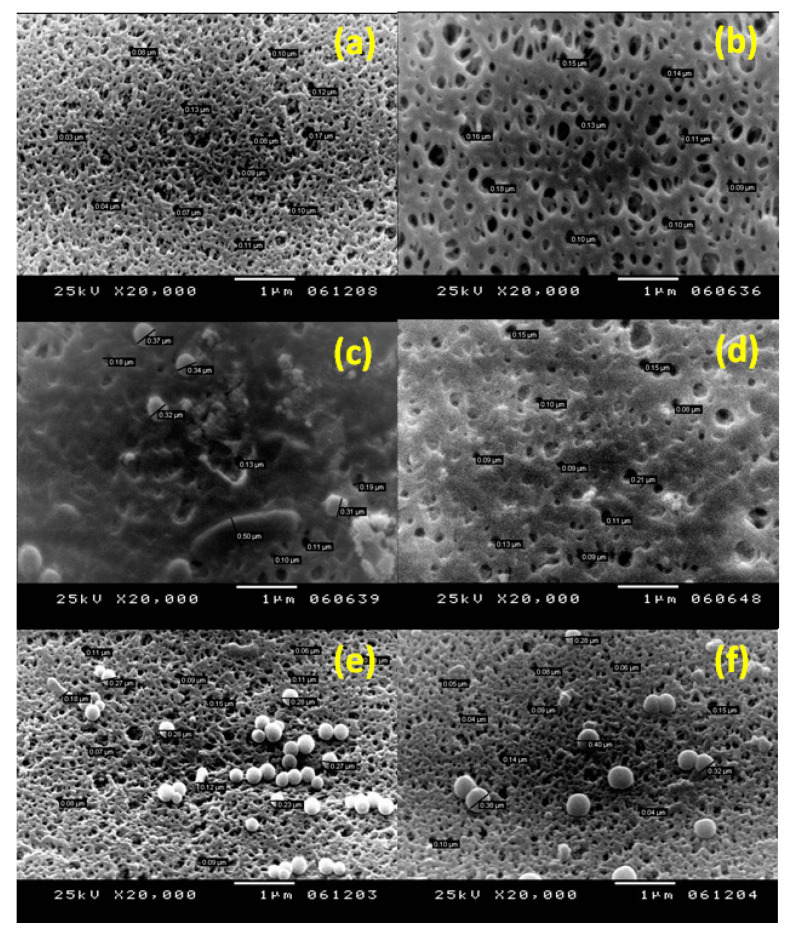
SEM images for unmodified PES membrane (**a**) and modified PES membrane (**b**) before filtration, respectively. SEM images of unmodified PES membrane (**c**) and modified PES membrane (**d**) after filtration of pretreated, inoculated feed seawater without the chlorination step, respectively. SEM images of unmodified PES membrane (**e**) and modified PES membrane (**f**) after filtration of pretreated, inoculated feed seawater pretreated with the chlorination step, respectively. Reference conditions: 1 bar, 23 ± 2 °C at 200 rpm stirring for 9 h (3 h × 3 days) filtration time. SEM images were taken at 20,000× magnification, and the scale bar is 1 µm.

Figure 12a,b show SEM images for unmodified PES membrane and modified PES membrane before filtration, respectively. For the feed water pretreated without chlorination, SEM images shown in Figure 12c,d show the formation of a thick layer of biofilm with EPS secretion and the complete blockage of most of the unmodified membrane pores. Meanwhile, the layer of biofilm formed on the modified membrane surface was not as thick as on the unmodified membrane and, clearly, the pores were not completely blocked. On the other hand, in the case of the chlorine disinfection step, the SEM image in Figure 12e shows the presence of bacteria on the unmodified membrane surface in aggregations at the start of biofilm formation, while, on the modified membrane surface, bacteria did not form aggregations. An SEM image (shown in Figure 12f) of the modified membrane surface under both conditions (i.e., chlorinated or non-chlorinated feed seawater) showed that bacteria were more loosely attached, as discussed before.

## 4. Conclusions

The filtration of pretreated, inoculated seawater using a modified PES UF membrane without the pre-chlorination step maintained the initial flux of the membrane as well as the largest permeated volume (productivity). The modified membrane was able to reject bacteria from the membrane surface in both the presence or absence of chlorine disinfectant. The addition of chlorine generally resulted in a cleaner membrane; however, its usage in conjunction with the modified membrane resulted in a significant reduction in the membrane flux. Furthermore, bacterial counts of chlorinated feedwater over three days of filtration reflected enhanced bacterial regrowth. SEM images showed a looser attachment of bacteria on the modified membrane surface.

In general, the modified PES membrane with a brush-like oligomer of the 3-AP modifier shows a higher membrane performance in terms of improving the quality and productivity of the filtrate as well as reducing the bacterial attachment onto the membrane’s surface. Both the steric hindrance and the osmotic effect of the hydrated brush-like polymer layer keep bacteria at a distance from the membrane surface, which facilitates their removal by routine membrane-washing procedures. On the other hand, the use of chlorine disinfectant in the pretreatment of feed water prior to UF had no evident effect; it resulted in a further reduction in both the quality and water productivity of the membrane compared to the modified one. Moreover, significant bacterial regrowth was enhanced by chlorine usage.

Depending on the obtained results from this study, many points still require further investigation to understand the effect of the membrane structure on the biofouling phenomenon. More studies are needed to investigate the effect of chlorination–dechlorination steps on the structure of the modifying layer. Moreover, the interaction between the modifying layer and the bacterial cells and its effect on biofilm formation require further in-depth studies.

## Supplementary Materials

The following are available online at https://www.mdpi.com/2077-0375/10/9/227/s1, Figure S1: Schematic representation of four possible chemical structure(s) of the PES surface after modification with 3-aminophenol (3-AP), containing O-linked and N-linked structures [47]. Figure S2: Photos of seawater (a) before coagulant addition, (b) after sedimentation, and (c) after pretreatment. Figure S3: Residual turbidity as a function of coagulant (FeCl_3_) concentration (mg·L^−1^ seawater).
