# Supplementary material for: Comparison between a Conventional Anti-Biofouling Compound and a Novel Modified Low-Fouling Polyethersulfone Ultrafiltration Membrane: Bacterial Anti-Attachment, Water Quality and Productivity
Source: Membranes (Basel). 2020 Sep 10;10(9):227. doi: 10.3390/membranes10090227 (PMC7558014; doi:10.3390/membranes10090227)
Supplement: Supplementary file 1 [file membranes-10-00227-s001.pdf]

Supplementary materials

Comparison between a Conventional Anti-Biofouling Compound and a Novel Modified Low-Fouling Polyethersulfone Ultrafiltration Membrane: Bacterial Anti-Attachment, Water Quality and Productivity

Norhan Nady [1,*], Noha Salem [2], Ranya Amer [3], Ahmed El-Shazly [4], Sherif H. Kandil [5], Mohamed Salah El- Din Hassouna [2]

[1] Polymeric Materials Research Department, City of Scientific Research and Technological Applications (SRTA-City), Borg El-Arab City 21934, Alexandria, Egypt.

[2] Department of Environmental Studies, Institute of Graduate Studies and Research, Alexandria University, Alexandria 21321, Egypt; nohasalem560@yahoo.com (N.S.), s.hassouna@gmail.com (M.S.E.-D.H.).

[3] Environment and Natural Material Research Institute (ENMRI), City of Scientific Research and Technological Applications (SRTA-City), New Borg El-Arab 21934, Alexandria, Egypt; ranyaamer@yahoo.com.

[4] Chemicals and Petrochemical Engineering Department, Egypt-Japan University of Science and Technology (E-JUST), Alexandria 21934, Egypt; ahmed.El-shazly@yahoo.com.

[5] Department of Materials Science, Institute of Graduate Studies and Research, Alexandria University, Alexandria 21321, Egypt; s.kandil@usa.net. (S.H.K.)

* Correspondence: norhan.nady77@yahoo.com; Tel.: +20-109-091-8521.

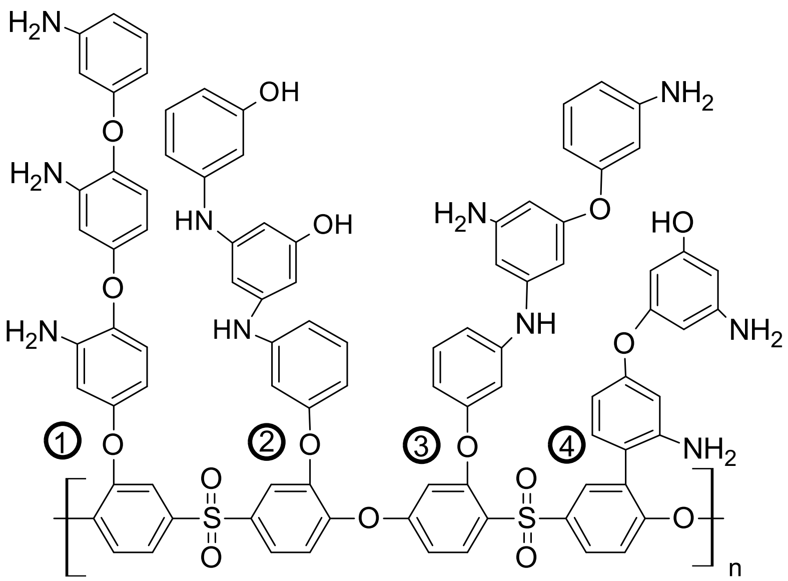

Figure S1. Schematic representation of four possible chemical structure(s) of the PES surface after modification with 3-aminophenol (3-AP), containing O-linked and N-linked structures [47].

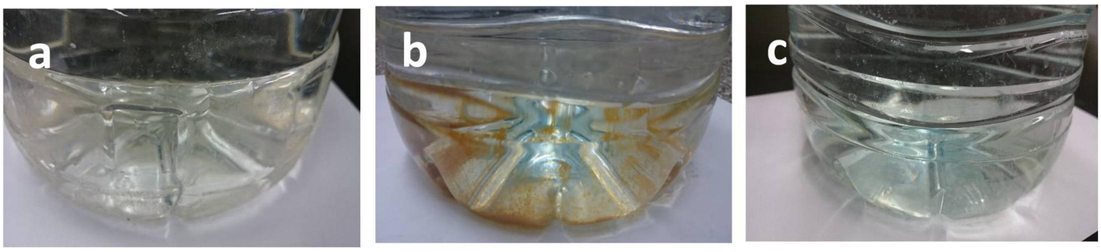

Figure S2. Photos of seawater (**a**) before coagulant addition, (**b**) after sedimentation, and (**c**) after pretreatment.

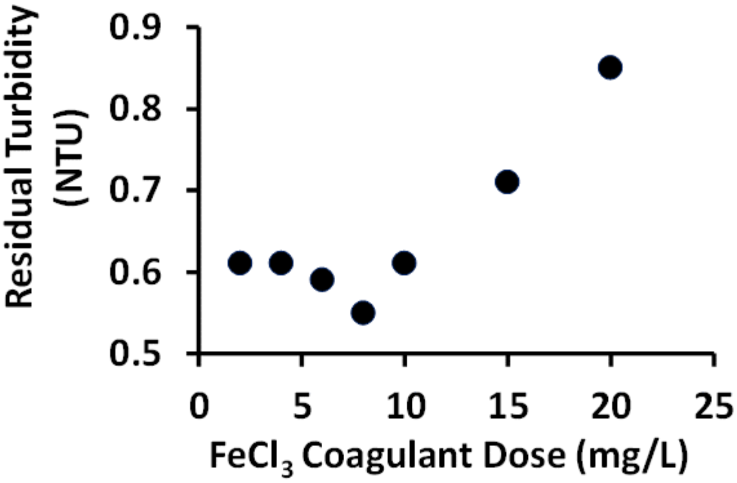

Figure S3. Residual turbidity as a function of coagulant ($FeCl_3$) concentration (mg L^{-1} seawater).
